# Premorbid characteristics of the SAPAP3 mouse model of obsessive-compulsive disorder: behavior, neuroplasticity, and psilocybin treatment

**DOI:** 10.1093/ijnp/pyaf022

**Published:** 2025-03-29

**Authors:** Michal Lazar, Michal Brownstien, Alexander Botvinnik, Chloe Shevakh, Orr Shahar, Tzuri Lifschytz, Bernard Lerer

**Affiliations:** Biological Psychiatry Laboratory and Hadassah BrainLabs, Center for Psychedelic Research, Hebrew University, Jerusalem, Israel; Biological Psychiatry Laboratory and Hadassah BrainLabs, Center for Psychedelic Research, Hebrew University, Jerusalem, Israel; Biological Psychiatry Laboratory and Hadassah BrainLabs, Center for Psychedelic Research, Hebrew University, Jerusalem, Israel; Biological Psychiatry Laboratory and Hadassah BrainLabs, Center for Psychedelic Research, Hebrew University, Jerusalem, Israel; Biological Psychiatry Laboratory and Hadassah BrainLabs, Center for Psychedelic Research, Hebrew University, Jerusalem, Israel; Biological Psychiatry Laboratory and Hadassah BrainLabs, Center for Psychedelic Research, Hebrew University, Jerusalem, Israel; Biological Psychiatry Laboratory and Hadassah BrainLabs, Center for Psychedelic Research, Hebrew University, Jerusalem, Israel

**Keywords:** self-grooming, head-body twitches, obsessive-compulsive disorder, SAPAP3, Tourette’s syndrome

## Abstract

**Background:**

SAPAP3-knockout (SAPAP3-KO) mice develop excessive self-grooming behavior at 4-6 months of age, serving as a model for obsessive-compulsive disorder (OCD). Given that anxiety often precedes OCD diagnosis in humans, this study investigated whether juvenile SAPAP3-KO mice exhibit anxiety-like behaviors before developing the self-grooming phenotype, and whether such behaviors respond to psilocybin (PSIL) treatment. The study also examined 4 key neuroplasticity-related synaptic proteins—GAP43, PSD95, synaptophysin, and SV2A—as SAPAP3 is a postsynaptic scaffold protein that interacts with PSD95 and may affect synaptic function.

**Methods:**

Two studies were conducted using male and female juvenile (10-13 weeks) SAPAP3-KO mice. Study 1 compared behavioral phenotypes between homozygous (HOM), heterozygous, and wild-type (WT) mice. Study 2 evaluated a different sample of HOM and WT mice and assessed the effect of PSIL (4.4 mg/kg) on identified behavioral differences. Both studies included comprehensive behavioral testing focused on anxiety-like behavior, social interaction, and cognitive function. Additionally, levels of 4 synaptic proteins were measured by western blots in the frontal cortex, hippocampus, amygdala, and striatum of juvenile and adult SAPAP3-KO mice.

**Results:**

In both studies, juvenile HOM SAPAP3-KO mice showed significant anxiety-like behaviors compared to WT mice, spending less time in open field center, and elevated plus maze open arms. They also buried fewer marbles and found fewer buried Oreos than WT mice. Psilocybin treatment did not improve these behavioral manifestations. Analysis of synaptic proteins revealed significant increases in GAP43, synaptophysin, and SV2A across multiple brain regions in adult male HOM mice and of SV2A in the frontal cortex of HOM females compared to WT, but not in juvenile mice of either sex.

**Conclusions:**

Juvenile SAPAP3-KO mice exhibit anxiety-like behaviors before developing the characteristic excessive self-grooming phenotype, paralleling the prodromal anxiety often seen in human OCD. Unlike in adult SAPAP3-KO mice, these manifestations were not responsive to PSIL treatment. The age-dependent increases in synaptic proteins observed in adult (but not juvenile) male SAPAP3-KO mice HOM for the deletion and to a lesser extent in female homozygotes, may represent compensatory plasticity changes in response to the phenotype. These results provide insights into the developmental trajectory of OCD-like behaviors and associated neuroplastic adaptations.

**Significance Statement**

Obsessive-compulsive disorder (OCD) frequently emerges during adolescence, with anxiety as a common prodromal symptom. This study investigated behavioral and molecular characteristics of juvenile SAPAP3 knockout (KO) mice aged 10-13 weeks, an established preclinical model of OCD, prior to manifestation of their characteristic excessive grooming phenotype. Juvenile SAPAP3-KO mice exhibited significant anxiety-like behaviors on multiple behavioral measures. While psilocybin treatment reduces OCD-like behaviors in adult SAPAP3 KO mice, it did not ameliorate anxiety-like behaviors in juvenile mice, indicating age-dependent therapeutic effects. Notably, adult male SAPAP3-KO mice showed elevated levels of synaptic plasticity-related proteins in emotion-regulatory brain regions, whereas juvenile KO showed no such alterations. These findings demonstrate that anxiety-like behavior precedes compulsive behaviors in this model and reveal age-dependent neuroplasticity changes. This developmental trajectory parallels clinical observations in OCD and provides a framework for investigating early intervention strategies.

## INTRODUCTION

Mice that carry a homozygous (HOM) deletion of the SAPAP3 gene manifest, from the age of 4-6 months, a characteristic phenotype consisting of repetitive bouts of self-grooming, head-body twitches, and anxiety-related behaviors.^1–3^ The SAPAP3 knockout mouse (SAPAP3-KO) is regarded by many investigators as a relevant preclinical model of obsessive-compulsive disorder (OCD) because of the resemblance of compulsive self-grooming to compulsive behaviors manifested by patients with OCD and OCD-related phenotypes such as trichotillomania and skin picking. The model thus has reasonable face validity (defined as the similarity of what is observed in the animal model to what is observed in the human-modeled organism).^4^ The self-grooming behavior and anxiety-like behavior of SAPAP3-KO mice are reduced by sub-chronic administration of the selective serotonin reuptake inhibitor, fluoxetine,^1^ which is used to treat OCD, reflecting a degree of predictive validity (the extent to which the performance of the animal model in response to a defined experimental manipulation correlates with or can predict the response of the human condition to that same independent variable).^4^ Moreover, there is evidence that suggests impaired cortico-striatal-thalamic connectivity in SAPAP3-KO mice, which was alleviated by ketamine administration.^5^ In patients with OCD, reduction of cortico-striatal hyperconnectivity was reported in patients who responded to treatment with dorsomedial prefrontal repetitive transcranial magnetic stimulation,^6^ suggesting that the SAPAP3-KO model of OCD may also have construct validity (a similarity in the biological underpinnings of the disease and the model).^4^ Although not definitively validated,^7^ the SAPAP3-KO model has increasing support as a model for OCD.

SAPAP3-knockout mice are reported to not manifest an excessive self-grooming phenotype before the age of 4-6 months.^1,2^ A key question is whether there are characteristic phenotypic manifestations in SAPAP3-KO mice prior to the emergence of the adult phenotype. Few studies have addressed this question directly. Tesdahl et al.^8^ recorded ultrasonic vocalizations from 5-day-old SAPAP3-KO mice and found an increase in the number and duration of these vocalizations compared to wild-type (WT) mice. Assessments of other behavioral manifestations in SAPAP3-KO mice under 4 months old are currently lacking. Thus, it is not clear whether anxiety-like manifestations observed in adult SAPAP3-KO mice are present before the emergence of the full phenotype. This is an important question because it is well-established that there is a significant comorbidity between anxiety and OCD.^9^ In this context, a recent study of 206 children and adolescents showed that generalized anxiety disorder was a significant predictor of obsessive-compulsive symptoms as well as risk for OCD.^10^ Early diagnosis of OCD is important because of the possibility of early intervention, which is a focus of considerable interest.^11^ Thus, phenotypic manifestations in SAPAP3-KO mice under 4 months old are a key unexplored question.

From the perspective of treatment, other than fluoxetine,^1^ there are reports that excessive self-grooming in SAPAP3-KO mice is reduced by a single administration of ketamine, the effect lasting for 3 days.^5^ The atypical antipsychotic, aripiprazole, was found to reduce head-body twitches and short grooming bouts in SAPAP3-KO mice but not long grooming bouts.^2^ There is growing interest in the potential of psychedelic drugs, particularly psilocybin (PSIL), to treat OCD.^12^ This interest is supported by case studies on patients with OCD,^13^ an open trial of PSIL in patients with OCD,^14^ and studies in mice using the marble burying (MB) test.^15,16^ In this context, it was recently demonstrated by Brownstien et al.^3^ that a single administration of PSIL or PSIL-containing psychedelic mushroom extract significantly reduced excessive self-grooming and also head-body twitches and anxiety-like manifestations in male and female SAPAP3-KO mice > 6 months old. The effect on self-grooming persisted for up to 42 days following a single administration.

In the current study, we examined male and female SAPAP3-KO mice aged 10-13 weeks for anxiety-related features and compared them to mice heterozygous (HET) for the mutation and WT mice. We then examined the effect of treatment with a single dose of PSIL on anxiety-related measures in a second cohort of juvenile (10-13 weeks) SAPAP3-KO mice. In addition, we examined the levels of 4 proteins that play a key role in synaptic plasticity in 4 different brain areas in juvenile and adult (>6 months) SAPAP3-KO mice. Our findings indicate that SAPAP3-KO mice manifest significant anxiety-related behavioral features before the emergence of the excessive self-grooming phenotype, but these manifestations are not responsive to treatment with PSIL. Significant increases in brain levels of neuroplasticity-related synaptic proteins were observed in adult (but not juvenile) SAPAP3-KO mice HOM for the deletion.

## MATERIAL AND METHODS

### Animals and Experimental Design

As previously described by Brownstien et al.,^3^ a SAPAP3-KO breeding colony was established using 5 HET SAPAP3-KO male and female mice kindly provided by Dr Guoping Feng (Massachusetts Institute of Technology). Mice were housed up to 8 per cage (cage size: 43 × 27 × 30 cm) under standardized conditions with a 12-h light/dark cycle, stable temperature (22 ± 1°C), controlled humidity (55 ± 10%), and free access to mouse colony chow and water. The cages included all genotypes under study. Throughout the experiment, male and female SAPAP3-KO mice were assessed separately in order to identify any significant difference in the behavioral phenotype between the sexes. The sample size for behavioral experiments was based on Brownstien et al.^3^ Experiments were approved by the Authority for Biological and Biomedical Models, Hebrew University of Jerusalem, Israel (Animal Care and Use Committee Approval Number: MD-21-16596-4). All efforts are made to minimize animal suffering and the number of animals used.

The purpose of Study 1 was to evaluate anxiety- and depression-like phenotypes, cognition, social interaction, and social dominance in SAPAP3 HOM, HET, and WT KO mice. Forty-four mice HOM for SAPAP3-KO were used for this study (20 male, 24 female), 42 HET mice (21 male, 21 female), and 55 WT mice (26 male, 29 female). Mice were aged 11.03 ± 0. 071 (mean ± SEM) weeks at the time of evaluation (Table S1). No treatments were administered to the mice in this study.

The purpose of Study 2 was to examine the effect of treatment with PSIL 4.4 mg/kg intraperitoneal (i.p.) on the performance of juvenile SAPAP3-KO mice on a series of tests that had shown significant differences between SAPAP3-KO genotypes in Study 1. For Study 2, 32 mice HOM for SAPAP3-KO were used (16 male, 16 female) and 32 WT mice (16 male, 16 female). HET mice were not used because the focus on treatment effects required clearly differentiated groups. Mice were 11.85 ± 0.87 (mean ± SD) weeks at the time of entry into the study and treatment administration (Table S1). Drugs were administered by i.p. injection in a standard injection volume in the ratio of 10 μL/1g 48 hours before behavioral tests were initiated. Within each cage, mice were randomly divided using a randomization table into the following treatment groups: vehicle (VEH): 0.9% saline (Male, *n* = 16; Female, *N* = 16); PSIL: 4.4 mg/kg dissolved in the saline VEH (Male, *n* = 16; Female, *N* = 16). Psilocybin was supplied by Usona Institute and was determined by AUC at 269.00 nm u**ltra** p**erformance** l**iquid** c**hromatography** (UPLC) to contain 98.75 wt.% PSIL. The dose of PSIL that we used (4.4 mg/kg) was chosen based on our previous dose-response study on the effect of PSIL on the head-twitch response^17,18^ and in view of the fact this dose is equivalent in mice to a 25 mg dose in humans according to the widely used DoseCal dose conversion methodology.^19^

### Genotyping

Following the “HOTSHOT” method, the genotype was determined by polymerase chain reaction of mouse tail DNA or by mouse ear hole DNA. Three different SAPAP3-KO primers are used to distinguish the genotypes of WT, HET, and HOM mice as follows: primer F1 (5′ ATTGGTAGGCAATACCAACAGG 3′) and Primer R1 (5′GCAAAGGCTCTTCATATTGTTGG 3′) identify WT allele (around 147 base pairs), while Primer F1 and TK F2 (5′ CTTTGTGGTTCTAAGTACTGTGG 3′) identify KO allele (around 222 base pairs).^1^

### Behavioral Tests

**Figure 1.**
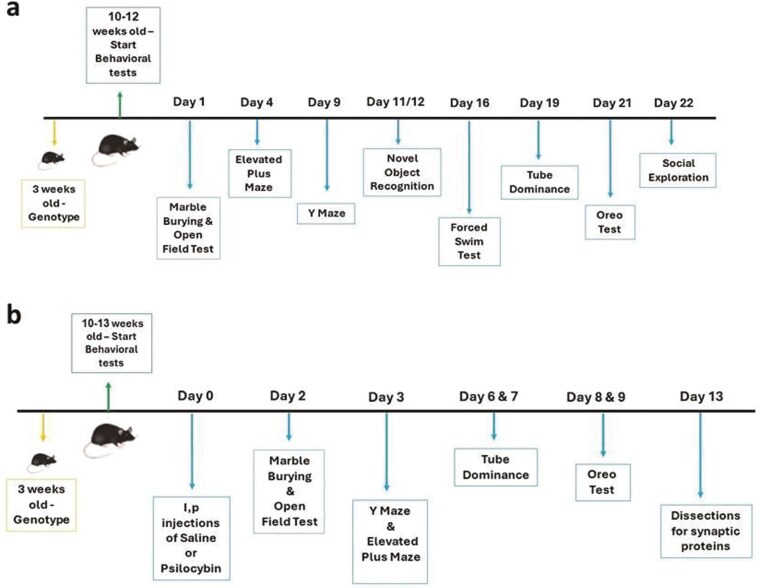
(A) Timeline of the behavioral test in Study 1. (B) Timeline of treatment and behavioral tests in Study 2.

For Study 1, behavioral tests were performed according to the schedule shown in Figure 1A. Since all the tests have objective outcome measures that are not dependent on subjective assessment, the experimenter was aware of the genotypes of the mice, but the statistical analysis was blind. The same experimenter (M.B.) performed all the behavioral assessments. Tests were performed between 9 AM and 4 PM each day, and mice were counterbalanced within each testing day and between testing days for sex and genotype. A detailed description of the tests is provided in the Supplementary Information section of this paper.

For Study 2, behavioral tests started 48 hours after drug administration, according to the schedule shown in Figure 1B. As in Study 1, the outcome measures were objective. Conditions of blinding, time of day for testing, and counterbalancing were the same as for Study 1, except that counterbalancing included treatment. M.L. performed all the assessments with the assistance of B.O. The methodology applied in the tests was the same as for Study 1, as described in Supplementary Information.

### Synaptic Protein Assays

#### Study 1

—Brain tissue samples (frontal cortex, hippocampus, amygdala, and striatum) were obtained, as previously described by Shahar et al.,^17^ from 60 adult SAPAP3-KO mice aged 7 months and 16 days ± 2.014 days (males) and 7 months and 26 days ± 1.936 days (females) (mean + SEM) (HOM: male *n* = 15, female *n* = 15; WT male *n* = 15, female *n* = 15) that had not undergone behavioral testing. Hippocampus and striatum have distinct and easily identifiable morphology and were dissected fully. The frontal cortex included all cortex brain matter sectioned at coronal bregma 0.26–2.1 mm and 0–3.5 mm dorsal to ventral by brain atlas.^20^ The amygdala included the brain matter sectioned at coronal bregma −1.22 to −2.18 mm, 4.8–6 mm dorsal to ventral, and 1.5–2.75 mm medial to lateral by brain atlas.^20^ Samples were stored at −80°C until assayed. Using Western blot analysis, we quantified 4 synaptic proteins (GAP43, PSD95, synaptophysin, and SV2A) in each of the 4 brain areas, as described in our previous report.^17^ Briefly, the brain tissue samples were lysed in Pierce radioimmunoprecipitation assay (RIPA) sample buffer (Thermo Fisher Scientific), supplemented with protease inhibitor cocktail (Roche Diagnostics), and boiled for 10 min. Equivalent amounts of protein extracts (20 mg) were analyzed by SDS–12% PAGE, followed by the transfer of the proteins to the polyvinylidene fluoride membrane. Blots were blocked in 5% fat-free milk in TBST buffer (Tris-Tween-buffered saline) and incubated in primary antibodies for 1 hour at room temperature. Primary antibodies included rabbit anti-GAP43 (ab75810, 1:2000; Abcam), rabbit anti-PSD95 (ab238135, 1:2000; Abcam), rabbit anti-Synaptophysin (ab32127, 1:2000; Abcam), rabbit anti-SV2A (ab54351, 1:100; Abcam), and mouse anti-β-Actin (8H10D10, 1:5000, Cell Signaling Technology). Blots were washed 3 times and incubated with horseradish peroxidase-conjugated secondary antibodies (1:5000, ABclonal) for 1 h, followed by repeated washing with TBST buffer. Proteins were visualized by using enhanced chemiluminescence (ChemiDoc Reader MP, Bio-Rad). The band intensity of each phosphorylated protein was normalized to the intensity of β-actin, which was used as a loading control (Representative immunoblots are shown in Figure S12A and B). Full blot images are shown in Figure S13.

#### Study 2

—Thirteen days after drug administration, brain tissue samples (frontal cortex, hippocampus, amygdala, and striatum) were obtained from 24 juvenile SAPAP3-KO mice aged 12.96 + 0.204 (mean ± SEM) weeks (HOM: male *n* = 5, female *n* = 6; WT male *n* = 7, female *n* = 6) SAPAP3-KO (*n* = 11) and WT mice (*n* = 13) that had been administered VEH, and were stored at −80°C. Synaptic proteins (GAP43, PSD95, synaptophysin, and SV2A) were quantified as described above (Representative immunoblots are shown in Figure S12C and D).

### Statistical Analysis

The experimental data are presented in all figures as the mean ±  SEM. To determine inter-group differences, student *t*-tests, or 1 or 2- or 3-way analysis of variance (ANOVA) were used. Analysis of covariance (ANCOVA) was performed if indicated. In Studies 1 and 2, Tukey’s tests were used to analyze post hoc comparisons. *P* < .05 (2-tailed) was the criterion for significance. Male and female mice were included in both studies,^21^ and results were tested for differences between the sexes by 2- (Study 1) or 3-way ANOVA (Study 2). Correlations were tested by the Pearson Product Moment Correlation Test. Graph Pad Prism, version 9.3.1 software was used for all statistical analyses. A nested *t*-test was performed to compare the expression levels of all 4 synaptic proteins (GAP43, PSD95, synaptophysin, SV2A) in each brain area (frontal cortex, hippocampus, amygdala, and striatum) between 2 genotypes (SAPAP3-KO and WT) and of each synaptic protein over all 4 brain areas. Statistical significance was assessed using a *t*-test (2-tailed). *P* < .05 was considered statistically significant. Results are shown as mean ±  SEM. Error bars in all figures represent SEM.

## RESULTS

### Behavioral Tests

#### Study 1

—In Study 1, SAPAP3 HOM, HET, and WT KO mice underwent a series of behavioral tests to evaluate anxiety- and depression-like phenotypes, cognition, social interaction, and social dominance. The schedule for these tests is given in Figure 1A. The results of this study show significant effects of the SAPAP3-KO genotype on several behavioral tests and no effect of sex.

**Figure 2.**
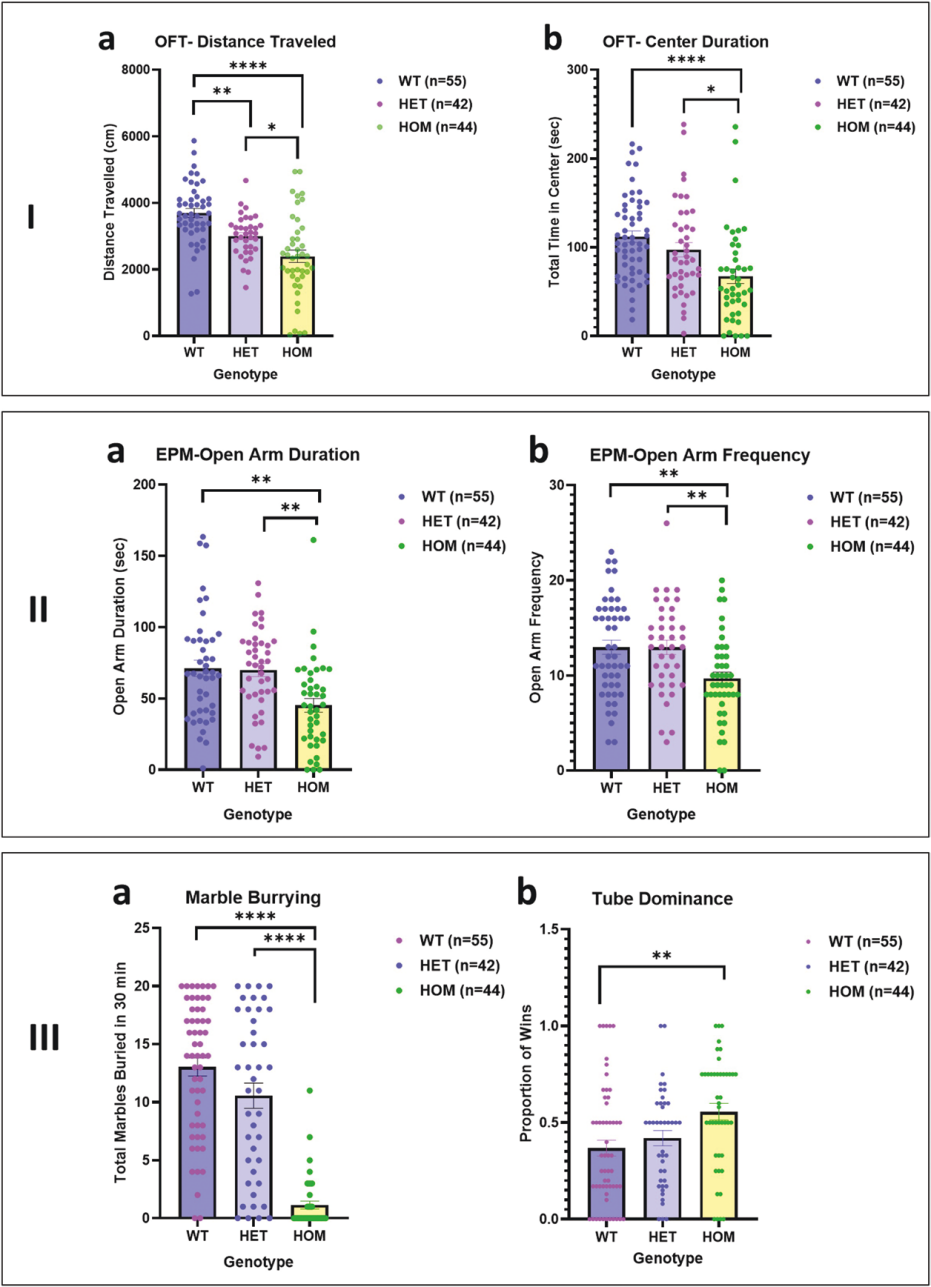
I. Open field test comparing wild type (WT), heterozygous (HET), and homozygous (HOM) SAPAP3 knockout (SAPAP3-KO) mice: (A) distance traveled (F_Genotype_ = 20.11, df = 2119, *P* < .0001). (B)Center duration (F_Genotype_ = 9.538, df = 2134, *P* = .0001). Tukey’s post hoc test: * *P* < .05, **P < .01, ****P < .0001. II. Elevated plus maze comparing WT, HET, and HOM SAPAP3-KO mice: (A) time spent in open arms (F_Genotype_ = 8.250, df = 2122 *P* = .0004). (B) Number of times entered open arms (F_Genotype_ = 6.943, df = 2127, *P* = .0014). Tukey’s post hoc test: **P < .01. III. –(A) Marble burying test, comparing WT, HET, and HOM SAPAP3-KO mice (F_Genotype_ = 60.93, df = 2134, *P* < .0001). Tukey’s post hoc test: ****P < .0001. –(B) Tube Dominance test, comparing WT, HET, and HOM SAPAP3-KO (F_Genotype_ = 5.489, df = 2136, *P* = .0051). Tukey’s post hoc test: **P < .01.

We focused on 2 key measures yielded by the open field test (OFT)—total distance traveled in the 10 minutes of the test and total time spent in the central area of the arena (Figure 2-IA and B). Two-way ANOVA showed a significant effect of genotype on total distance traveled (F = 20.11, df = 2, 119, *P* < .0001) but no significant effect of sex and no sex by genotype interaction. Scores of HOM mice were significantly lower than those of WT (*P* < .00 01) and HET mice (*P* < .05), and HET SAPAP3-KO mice traveled a significantly lower distance (*P* < .01) than WT mice. For time spent in the center of the open field, 2-way ANOVA showed a significant effect of genotype (F = 9.54, df = 2, 134, *P* = .0001) but no significant effect of sex or sex by genotype interaction. Homozygous mice spent significantly less time in the center than WT (*P* < .0001) and HET mice (*P* < .05).

On the elevated plus maze (EPM), time spent in the unprotected open arms and frequency of entry into these arms are indicative of the level of anxiety-like features with mice showing more anxiety-like behavior spending less time in these arms and entering less frequently. Two-way ANOVA showed a significant effect of genotype but not sex on time spent in the open arms (F = 8.25, df = 2, 122, *P* = .0004) and frequency of entry into the open arms (F = 6.943, df = 2127, *P* = .0014) (Figure 2-IIA and B). Homozygous mice spent less time in the open arms than WT and HET mice and entered the open arms less frequently (*P* < .01). To ensure that the EPM findings were not a consequence of lower activity of the HOM mice, we performed an ANCOVA entered distance covered on the EPM as a covariate. There was a non-significant effect of distance traveled on the EPM (F = 1.69, df = 1, 119, *P* > .10) and a significant effect of genotype (F = 9.61, df = 2, 119, *P* < .001). For frequency of entry into the open arms, there was a similar non-significant effect of distance traveled on the EPM (F = .−1, df = 1, 119, *P* > .10) and a significant effect of genotype (F = 5.23, df = 2, 119, *P* < .01).

On the MB test (Figure 2-III), the key measure is the number of marbles buried over 30 minutes. Two-way ANOVA showed a significant effect of genotype on number of marbles buried (F = 60.13; df = 2, 134, *P* < .0001) but no significant effect of sex or sex by genotype interaction (Figure 2-IIIA). Homozygous mice buried significantly fewer marbles than WT and HET mice (*P* < .000 1). To determine whether lower MB in the SAPAP3-KO mice could be related to the activity level of the mice, we examined the correlation between the number of marbles buried and the distance covered in the OFT. The correlation was significant (r = 0.35, *P* < .0001, 140 XY pairs), indicating that mice that are less active bury fewer marbles and vice versa. We further examined whether MB could be related to the level of anxiety-like manifestations of the SAPAP3-KO mice as reflected in the time spent in the open arms of the EPM. This correlation was also significant (r = 0.22, *P* = .007, 140 XY pairs), indicating that mice manifesting more anxiety-like features bury fewer marbles. There was a trend for distance covered in the OFT and time spent in the open arms of the EPM to be related, but this was not significant (r = 0.27, *P* = .07, 140 XY pairs).

Performance on the tube dominance test reflects social assertiveness as reflected in the proportion of wins.^22^ Two-way ANOVA showed a borderline significant effect of genotype on winning (F = 5.48, df = 2, 136, *P* = .05) but no significant effect of sex or sex by genotype interaction. On post hoc testing (Tukey), HOM mice achieved a significantly higher win proportion than WT mice (*P* < .01).

The Oreo test is based on the preference of mice for sweet objects.^23^ The first assessment was whether non-food restricted, previously habituated mice placed in a cage with a buried Oreo would find the cookie, dig it up, and eat it. There was a significant effect of genotype on this parameter among male mice (Table 1). Among them 88.96% of male WT mice found the Oreo and 81.82% of male HET mice but only 35.00% of male HOM mice (χ^2^ = 16.75, df = 2, *P* = .0002). Among female mice, the differences were not significant (WT 62.07% found; HET 61.90% found; HOM 58.33% found; χ^2^ = 0.10, df = 2, *P* = .93). There was no significant effect of genotype on Oreos eaten by those mice that found them among males or female mice (Table 1a). The number of Oreos found and number of Oreos eaten were not related to activity level (distance traveled on the OFT) (r = 0.07, *P* > .1, 140 XY pairs) nor to level of anxiety-like features (time spent in the open arms of the EPM) (r = 0.07, *P* > .1, 140 XY pairs).

**Table 1.** Effect of SAPAP3 knockout (SAPAP3-KO) genotype on the likelihood of mice carrying the different genotypes—WT (wild type), HET (heterozygous), and HOM (homozygous)—finding and eating Oreo cookies buried in the cage (the number and percent of mice that carrying each genotype that found cookies in the test and number of those which found cookies that ate them are given)

a. Study 1				
	No.	Percent	χ^2^ (df)	Significance
*Male*				
Found Oreos WT	20/23	88.96		
HET	18/22	81.82		
HOM	7/20	35.00	16.75 (2)	*P* = −.0002
Ate Oreos WT	8/20	40.00		
HET	6/18	33.33		
HOM	4/7	57.14	1.25 (2)	*P* = .54
*Females*				
Found Oreos WT	18/29	62.07		
HET	13/21	61.90		
HOM	14/24	58.33	0.10 (2)	*P* = .93
Ate Oreos WT	9/18	50.00		
HET	10/13	76.92		
HOM	9/14	64.29	2.49 (2)	*P *= .28

b. Study 2				
	No.	Percent	χ^2^ (df)	Significance
*Saline-treated* *males*				
Found Oreos WT	8/8	100.00		
HOM	4/8	50.00	5.33 (1)	*P* < .05
Ate Oreos WT	8/8	100.00		
HOM	1/4	25.00		*P* > .1 (Fisher)
*Females*				
Found Oreos WT	7/8	87.50		
HOM	2/8	25.00	7.27 (1)	*P* < .01
Ate Oreos WT	6/7	85.71		
HOM	1/3	33.33		*P* > .1 (Fisher)
*Psilocybin-treated* *male*				
Found Oreos WT	7/8	87.5		
HOM	2/8	25.00	7.27 (1)	*P* < .01
Ate Oreos WT	6/7	85.71		
HOM	1/2	50.00		*P* > .10 (Fisher)
*Females*				
Found Oreos WT	8/8	100.00		
HOM	3/8	37.50	8.73 (1)	*P* < .01
Ate Oreos WT	7/8	88.89		
HOM	1/3	33.33		*P* > .10 (Fisher)

Contrary to these positive findings, other tests did not show an effect of the SAPAP3-KO genotype. On the forced swim test (FST), there was no significant effect of genotype on duration (Figure S1a) or frequency (Figure S1b) of inactivity in mice. The social exploration test (Figure S2a) and the NOR test (Figure S2b and c) also showed no significant differences between genotypes.

#### Study 2

—In Study 2, we examined the effect of treatment with PSIL 4.4 mg/kg i.p. on the performance of juvenile SAPAP3-KO mice (mean age 11.85 ± 0. 213 weeks) on a series of tests that had shown significant differences between SAPAP3-KO genotypes in Study 1. The study timeline and test battery are summarized in Figure 1B.

There were no significant effects of PSIL treatment on any of the behavioral measures, as indicated by the lack of a significant main effect of treatment on 3-way ANOVA. There was also no significant effect of sex on any of the behavioral measures. However, there were significant effects of genotype over and above treatment and sex and significant post hoc effects in both saline and PSIL-treated mice on a number of tests.

On the OFT (Figure 2-IA and B), which examines activity and anxiety-like behaviors, 3-way ANOVA showed a significant effect of genotype on total distance traveled (F = 156.00, df = 1, 56, *P* < .0001) but no significant effect of sex or treatment with PSIL. Distance traveled by HOM mice was significantly lower than that covered by WT in both the saline (*P* < .001) and the PSIL (*P* < .001) groups. Homozygous mice spent significantly less time in the open arms of the EPM than WT mice in both the saline (*P* < .0001) and the PSIL (*P* < .001) groups. For Center Duration, there was a significant effect of genotype (F = 11.37, df = 1, 66, *P* = .0013) but on post hoc testing, the difference was significant in the PSIL group only (*P* < .01).

On the EPM, 3-way ANOVA showed a significant effect of genotype on total distance traveled (F = 50.97, df = 1, 56, *P* < .0001) but no significant effect of sex or treatment. Distance traveled by HOM mice was significantly lower than that covered by WT in both the saline (*P* < .001) and the PSIL (*P* < .0001) groups. The effect of genotype was also significant for the frequency of entry into the open arms (F = 24.42, df = 1, 67; *P* < .0001). Elevated plus maze frequency of entry into the open arms was significantly lower in the HOM than the WT mice for both VEH (*P* < .05) and PSIL-treated mice (*P* < .0 1). As in Study 1, we determined the contribution of distance covered on the EPM to these results. For duration in the open arms, ANCOVA showed a non-significant effect of distance traveled on the EPM (F = 1.69, df = 1, 119, *P* > .10) and a significant effect of genotype (F = 9.61, df = 2, 119, *P* < .001) for duration in the open arms. For frequency of entry into the open arms, there was a similar non-significant effect of distance traveled on the EPM (F = 3.52, df = 1, 58, *P* > .05) and a significant effect of genotype (F = 8.19, df = 1, 58, *P* < .01).

**Figure 3.**
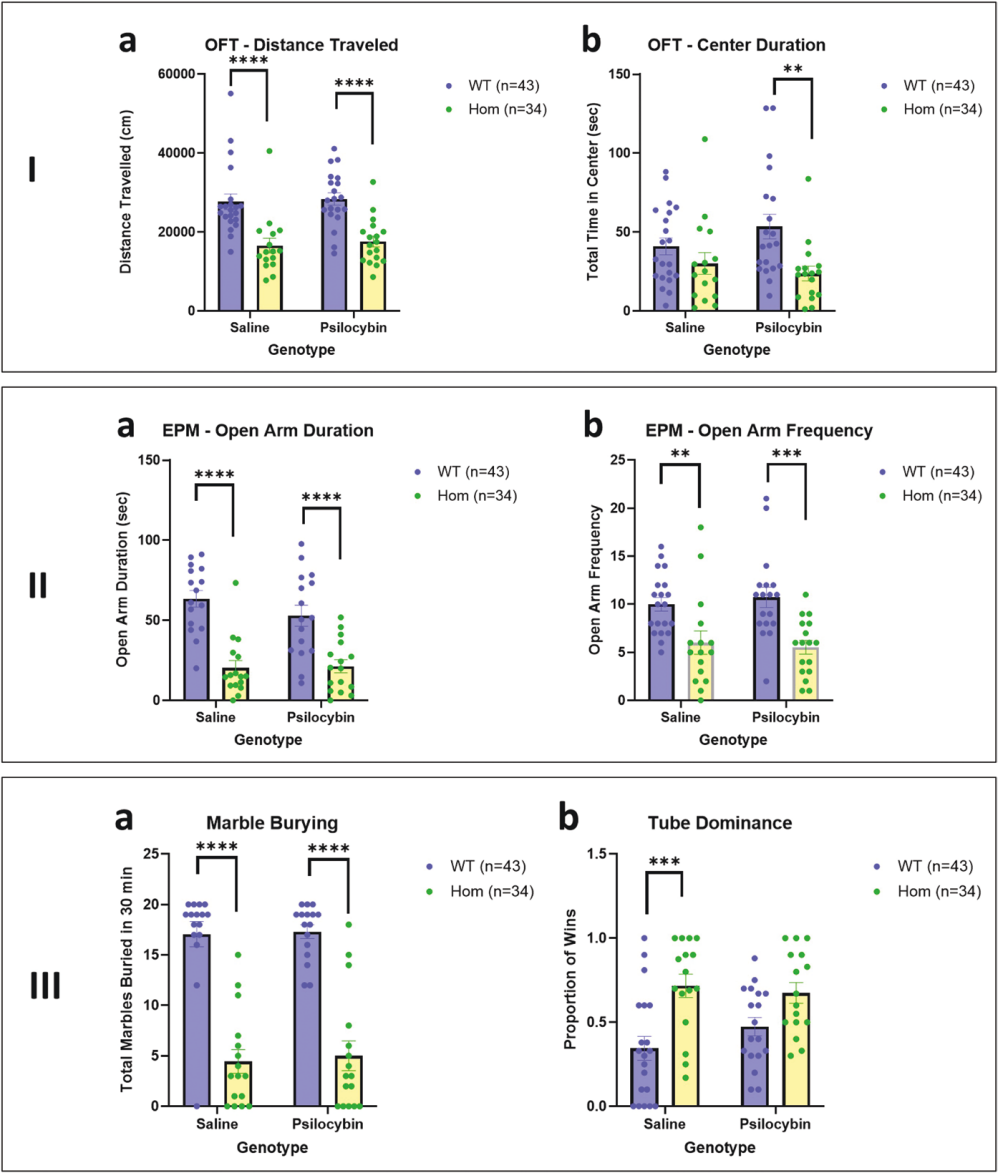
I. Open field test comparing WT and HOM SAPAP3-KO mice with and without treatment with psilocybin: (a) Distance traveled (F_Genotype_ = 39.31, df = 1, 67, *P* < .0001). (b) Center duration (F_Genotype_ = 11.37, df = 1, 66, *P* = .0013).** *P* < .01, **** *P* < .0001 (Tukey’s post hoc test). II. Elevated plus maze test comparing WT and HOM SAPAP3-KO mice with and without treatment with psilocybin: (a) open arms duration (F_Genotype_ = 50.97, df = 1, 56, *P* < .0001). (b) Open arm frequency (F_Genotype_ = 24.42, df = 1,67; *P* < .0001). ** *P* < .01, **** *P* < .0001 (Tukey’s post hoc test). III. (a) Marbles buried in 30 minutes comparing WT and HOM SAPAP3-KO mice with and without treatment with psilocybin (F_Genotype_ = 116.2, df = 1, 56, *P* < .0001). (b) Tube Dominance Test in WT and SAPAP3-KO mice, with our without treatment with psilocybin (F_Genotype_ = 18.76, df = 1,62, *P* < .0001). * *P* < .05, *** *P* < .001, **** *P* < .0001 (Tukey’s post hoc test). HET, heterozygous; HOM, homozygous; SAPAP3-KO, SAPAP3 knockout; WT, wild type.

As in Study 1, the MB test (Figure 3-III) yielded striking results. Three-way ANOVA showed a significant effect of genotype on number of marbles buried (F = 116.20, df = 1, 56, *P* < .0001) but no significant effect of sex or treatment. Both saline and PSIL-treated HOM mice buried significantly fewer marbles than WT mice (*P* < .0001). As in Study 1, we sought to determine whether lower MB in the SAPAP3-KO mice could be related to the activity level of the mice. We examined the correlation between the number of marbles buried and the distance covered in the OFT. The correlation was significant (r = 0.45, *P* = .0002, 64 XY pairs); mice that covered less distance of the OFT buried fewer marbles and vice versa. We further examined whether lower MB could be related to the level of anxiety-like behavior of the SAPAP3-KO mice as reflected in the time spent in the open arms of the EPM. This correlation was also significant (r = 0.57, *P* = <.0001, 54 XY pairs); mice that spent less time in the open arms of the EPM buried fewer marbles and vice versa. It is noteworthy that distance traveled on the OFT and time spent in the open arms of the EPM were correlated (r = 0.52, *P* < .0001, 54 XY pairs), that is, greater activity was associated with less anxiety-like behavior and vice versa.

On the Tube Dominance test (Figure 3-IVB), three-way ANOVA showed a significant effect of genotype on the proportion of wins (F = 18.70, df = 1,62, *P* < .0001) but no significant effect of sex or treatment. Saline-treated HOM mice achieved a significantly higher proportion of wins than WT mice (*P* < .001). We examined the correlation between the proportion of wins and the distance covered in the OFT. The correlation was significant but inverse (r = −0.42, *P* = .0003, 70 XY pairs), that is, mice that won more frequently covered less distance on the OFT and vice versa. We further examined whether the proportion of wins could be related to the level of anxiety-like manifestations of the SAPAP3-KO mice as reflected in the time spent in the open arms of the EPM. This correlation was also significant and inverse (r = −0.31, *P* = .013, 63 XY pairs), indicating that mice with fewer anxiety-like features (more time in the open arms of the EPM) have a higher proportion of wins and vice versa.

The Oreo test results in Study 2 (Table 1b) were similar to those in Study 1. Whether treated with saline or PSIL, WT mice found the Oreo cookie significantly more frequently than HOM mice. The percentage of mice succeeding was > 85% in all cases. For HOM mice, the percentage finding the Oreo was 50% or less. Comparisons between WT and HOM for Oreos were statistically significant for both sexes. For Oreos eaten, there was a clear numerical trend for WT mice to eat more of the Oreos found, but the number of observations was too small to permit statistical significance to be demonstrated. We examined the correlation between the percentage of Oreos found and the distance covered in the OFT. The correlation was significant (r = 0.41, *P* = .0007, 64 XY pairs), that is, mice that were more active found more Oreos. We further examined whether the percentage of Oreos found could be related to the level of anxiety-like behavior of the SAPAP3-KO mice as reflected in the time spent in the open arms of the EPM. This correlation was also significant (r = 0.52, *P* < .0001, 59 XY pairs), that is, mice that were less anxious (more time in the open arms of the EPM) found more Oreos.

### Synaptic Proteins

#### Study 1

**Figure 4.**
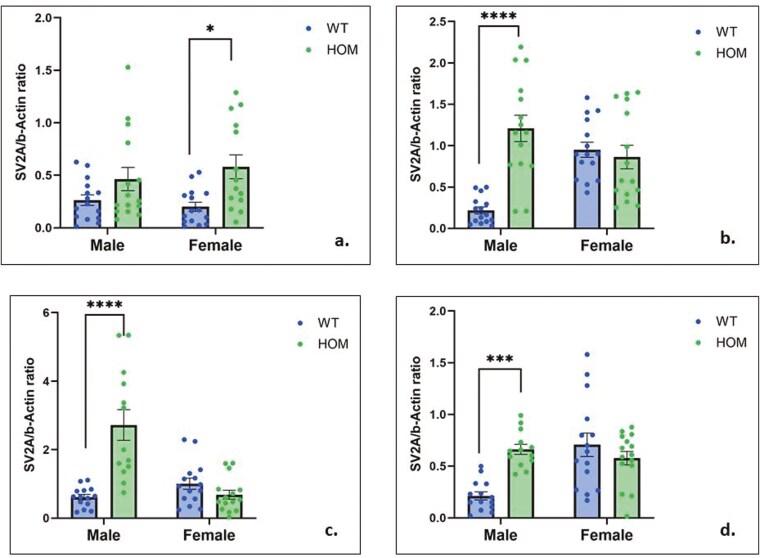
Study 1: 2-way analysis of variance (ANOVA) of SV2A levels in the: (a) frontal cortex: F_Genotype_ = 17.22, df = 1,55, *P* = .001; (b) hippocampus: F_Genotype_ = 14.67, df = 1,56, *P* = .0003; F_Genotype × Sex_ = 20,94, df = 1,56, *P* = .00; (c) amygdala: F_Genotype_ = 14.48, df = 1,53, *P* = .0004; F_Sex_ = 12.32, df = 1,53, *P* = .0009; F_Genotype × Sex_ = 26.75, df = 1,53, *P* < .0001; (d) striatum: F_Genotype_ = 4.59, df = 1,53, *P* = .03; F_Sex_ = 7.48, df = 1,53, *P* = .008; F = 15.08, df = 1,53, *P* = .0003. * *P* < .05, *** *P* < .001, **** *P* < .0001 (Tukey’s post hoc test).

—Effects of sex and genotype on levels of 4 synaptic proteins (GAP43, PSD95, synaptophysin, and SV2A) were analyzed in the 4 brain areas we studied (frontal cortex, hippocampus, amygdala, and striatum) by 2-way ANOVA (see Figure 4, Figures S3-S5). The synaptic protein that showed the strongest effect of genotype was SV2A (Figure 4). Two-way ANOVA showed a significant effect of genotype on SV2A levels in the frontal cortex (F = 17.22, df = 1,55, *P* = .001), with female HOM mice manifesting significantly higher SV2A levels than female WT mice (Tukey post hoc, *P* = .01). Genotype effects were also highly significant for SV2A in the hippocampus (F = 14.67, df = 1,56, *P* = .0003), amygdala (F = 14.48, df = 1,53, *P* = .0004), and striatum (F = 4.59, df = 1,53, *P* = .03). Post hoc tests showed that SV2A levels were significantly higher in male HOM than male WT mice in all 3 areas (*P* < .0001).

Significant effects of genotype were also observed for GAP43 in the frontal cortex (F = 7.30, df = 1,56, *P* = .009) and amygdala (F = 8.24, df = 1,54, *P* = .005); post hoc significance for male HOM vs male WT was 0.02 and 0.04 for each area, respectively (Figure S3). For PSD95, there was a significant effect of genotype in the frontal cortex (F = 6.70, df = 1,54, *P* = .01) (Figure S4), and for synaptophysin in the amygdala (F = 7.34, df = 1,56, *P* = .008), but post hoc tests were not significant in either sex separately (Figure S5).

There were significant sex effects on SV2A levels in the amygdala (F = 12.32, df = 1,53, *P* = .0009) and striatum (F = 7.48, df = 1,53, *P* = .008) (Figure 4); on GAP43 in the amygdala (F = 6.15, df = 1,54, *P* = .01) (Figure S3); on PSD95 in the frontal cortex (F = 5.43, df = 1,54, *P* = .02) and hippocampus (F = 4.38, df = 1,56, *P* = .04) (Figure S4); and on synaptophysin in the striatum (F = 11.77, df = 1,55, *P* = .001) (Figure S5). A significant sex × genotype interaction was observed for SV2A in the hippocampus (F = 20,94, df = 1,56, *P* = .0001), amygdala (F = 26.75, df = 1,53, *P* < .0001), and striatum (F = 15.08, df = 1,53, *P* = .0003) (Figure 4) and for synaptophysin the frontal cortex (F = 13.51, df = 1,55, *P* = .005) (Figure S5).

**Figure 5.**
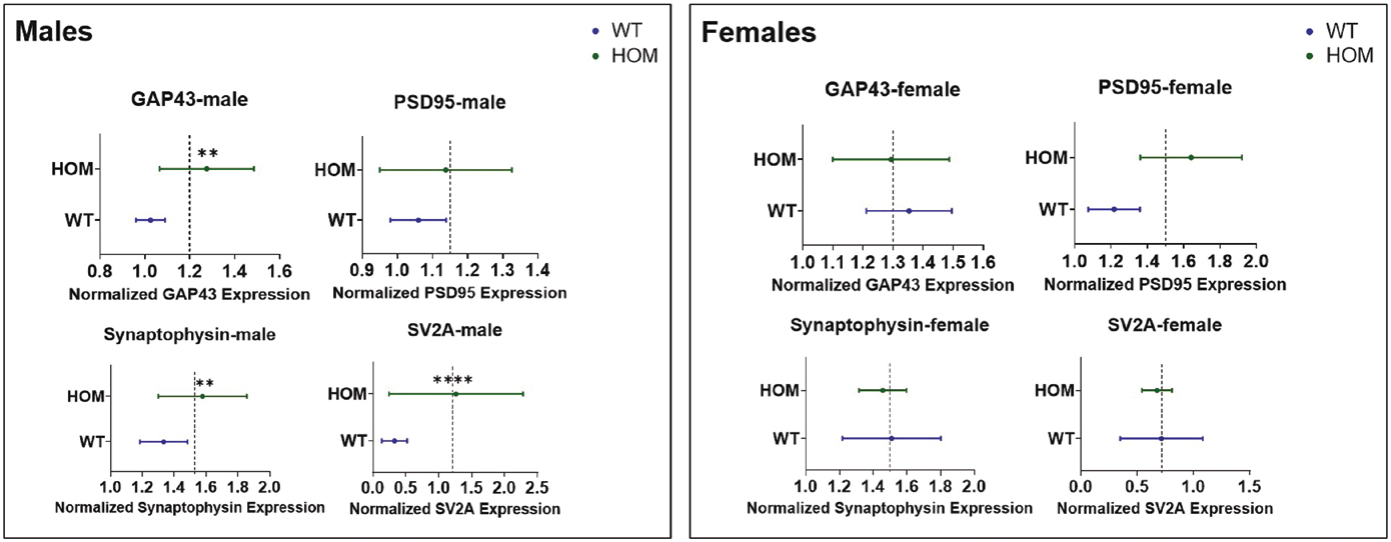
Study1: nested analysis of synaptic proteins (GAP43, PSD95, synaptophysin, and SV2A) by genotype (SAPAP3-KO and WT) across 4 brain areas (frontal cortex, hippocampus, amygdala, and striatum) in male (left) and female (right) mice. The X axis represents normalized synaptic protein expression levels across genotypes (Y axis). The dotted line shows the mean of the highest and lowest expression values between HOM and WT groups. Statistical significance was assessed using a *t*-test (2-tailed). Results are shown as mean ± SEM. ** *P* < .01, **** *P* < .0001. HOM, homozygous; SAPAP3-KO, SAPAP3 knockout; WT, wild type.

**Figure 6.**
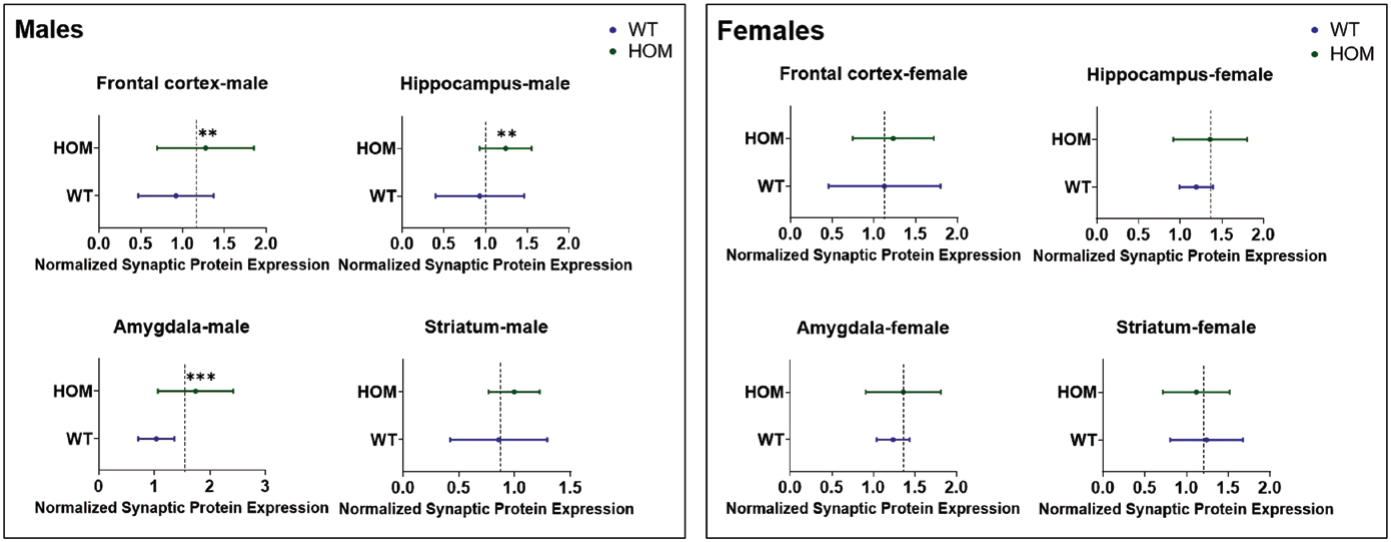
Study 1: nested analysis of 4 synaptic proteins (GAP43, PSD95, synaptophysin, and SV2A) within each of 4 brain areas (frontal cortex, hippocampus, amygdala, striatum) in male (left) and female (right) HOM vs WT SAPAP3-KO mice. The X axis represents normalized synaptic protein expression levels across genotypes (Y axis). The dotted line shows the mean of the highest and lowest expression values between the HOM and WT groups. Statistical significance was assessed using a *t*-test (2-tailed). Results are shown as mean ± SEM. ** *P* < .01, *** *P* < .001. HOM, homozygous; SAPAP3-KO, SAPAP3 knockout; WT, wild type.

To analyze synaptic proteins over all 4 brain areas, nested *t*-tests were performed (Figure 4) comparing each synaptic protein in HOM and WT mice over all brain areas. Nested *t*-tests were performed separately for male and female mice. In males, the analysis showed significantly higher levels of GAP43 (*P* = .001), synaptophysin (*P* = .003), and SV2A (*P* < .0001) but not of PSD95 across the 4 brain areas in HOM vs WT SAPAP3-KO mice (Figure 4, left panel). None of the differences were significant for female SAPAP3-KO mice (Figure 5, right panel). Examining all 4 synaptic proteins within each brain area separately, significantly higher synaptic protein levels were observed in male HOM SAPAP3-KO mice than in WT mice in the frontal cortex (*P* = .01), hippocampus (*P* = .01), and amygdala (*P* = .001) but not striatum (Figure 6, left panel). No significant differences were observed in any of the brain areas in female mice (Figure 6, right panel).

#### Study 2

We analyzed synaptic protein neuroplasticity markers on brain samples obtained from 11 HOM (5 males and 6 females for each genotype) and 13 WT (7 males and 6 females for each genotype) VEH-treated mice on day 13 after treatment administration. PSIL-treated mice were not examined. We performed 2-way ANOVA for each of the 4 synaptic proteins in each of the 4 brain areas, with sex and genotype as factors (Figures S6-S9). This analysis revealed a significant sex effect for PSD95 in the amygdala (F = 7.10, df = 1,16, *P* = .01), a significant sex by genotype interaction for PSD95 in the hippocampus (F = 7.54, df = 1,20, *P* = .01) and a significant sex by genotype interaction for synaptophysin in the striatum (F = 7.49, df = 1,20, *P* = .008). No post hoc comparisons of HOM vs WT genotype in males or females were significant for any of the significant sex effects or sex × genotype interactions. We performed a nested *t*-test comparing each synaptic protein (GAP43, PSD95, synaptophysin, SV2A—Figure S10) in HOM vs WT mice over all brain areas (frontal cortex, hippocampus, amygdala, and striatum). This analysis did not show significant differences between genotypes for any of the synaptic proteins across all brain areas. For analysis of the synaptic protein neuroplasticity markers within each brain area, a nested *t*-test was performed comparing levels of all 4 synaptic proteins in HOM and WT mice in each brain area (frontal cortex, hippocampus, amygdala, and striatum—Figure S11). This analysis did not show significant differences over all synaptic proteins within any of the brain areas.

## DISCUSSION

Age of onset in OCD has been reported to be bimodal, with a mean of 12.8 (SD = 4.9) years in the early-onset group and 24.9 (SD = 9.3) years in the late-onset group.^24^ Anxiety is a common comorbid feature of OCD,^8^ and prodromal anxiety symptoms are frequent in youth^10^ and adults^25^ who subsequently develop OCD. Since the excessive self-grooming observed in SAPAP3-KO mice is regarded as a possible rodent model of OCD-like behavior, it is of interest to know whether SAPAP3-KO mice below the age at which the characteristic phenotype is observed manifest behavioral and other abnormalities. This question was the focus of the current study in which SAPAP3-KO mice of both sexes were assessed for a series of behavioral characteristics, including anxiety- and depressive-like features, cognitive function, sociability, and social dominance. The effect of treatment with PSIL on these behavioral features was evaluated. In addition, we measured levels of synaptic proteins in 4 brain regions in mice HOM for the SAPAP3 deletion compared to WT mice as markers of neuroplasticity.^17^

Our results in Study 1 for time spent in the center of the open field and time spent in the open arms of the EPM show clear evidence for higher levels of anxiety-like behavior in in male and female HOM SAPAP3-KO mice compared to WT mice. These findings were supported by those of Study 2, for females in regard to center duration in the open field and for both sexes in regard to time spent in the open arms of the EPM. In both studies, time spent in the open arms of the EPM was not related to activity reflected in the distance covered on the EPM. This evidence for more anxiety-like features in juvenile SAPAP3-KO mice is in accordance with findings of Welch et al.^1^ and Brownstien et al.^3^ in adult SAPAP3-KO mice that manifest the full self-grooming and head-twitch phenotype. Welch et al.^1^ found that 6 days of treatment with fluoxetine significantly alleviated anxiety-like features on the EPM. Brownstien et al.^3^ found that a single treatment with psychedelic mushroom extract (containing 4.4 mg/kg PSIL) significantly improved anxiety-like behavior on the OFT and EPM; treatment with chemical PSIL at the same dose was not as effective. In Study 2 of the current project, PSIL 4.4 mg/kg was not effective in improving anxiety-like behavior in SAPAP3-KO mice. The effect of psychedelic mushroom extract remains to be studied in juvenile SAPAP3-KO mice.

The MB test showed intriguing and highly significant effects of genotype in both sexes. In Study 1 and Study 2, juvenile SAPAP3-KO mice HOM for the deletion buried significantly fewer marbles over 30 minutes than WT mice. Similar findings were reported by Brownstien et al.^3^ in adult SAPAP3-KO mice. Digging, burrowing, and burying are natural behaviors in rodents and not necessarily indicative of pathology.^26^ Nevertheless, the MBT is widely used as a predictive test for the potential efficacy of compounds in treating OCD^15,27^ and as a measure of anxiolytic effects.^28^ However, the relevance of the test has been questioned on the grounds of face and predictive validity^29,30^ In the current study, reduced marble burying in SAPA2-KO mice was related to a lower level of activity and a higher level of anxiety-like behavior, as indicated by significant correlations between the number of marbles buried and activity on the OFT and time spent in the open arms of the EPM, respectively.

In adult SAPAP3 KO mice treated with a single injection of PSIL (4.4 mg/kg) or psychedelic mushroom extract containing the same dose of PSIL, MB was significantly increased by the treatment.^3^ The MBT was performed by Brownstien et al.^3^ 3 days after the administration of the mushroom extract. At this time, the pathological self-grooming and head-body twitches characteristic of adult HOM SAPAP3-KO mice were also alleviated by psychedelic mushroom extract and by PSIL. In contrast to the effect of PSIL on MB in adult SAPAP3-KO mice, in Study 2 of the current project, there was no effect of PSIL on the same phenotype in juvenile mice. This suggests that the effect of PSIL on behavioral manifestations of SAPAP3 deletion may be related to the maturation of brain systems. Brain maturation, especially in circuits implicated in compulsive behaviors or OCD-like symptoms, may influence PSIL’s efficacy. Psilocybin interacts with serotonin receptors which are known to play a critical role in mood and behavior. During maturation, the distribution, density, and sensitivity of these receptors undergo significant changes, potentially affecting how PSIL modulates neural activity.^31^ In adult SAPAP3-KO mice, mature receptor networks and more developed neural circuits may provide a suitable substrate for PSIL’s action, particularly in areas like the prefrontal cortex or amygdala, which are crucial in regulating compulsive behavior. The synaptic plasticity and connectivity patterns in a mature brain could allow PSIL to exert more profound effects on neural circuit function, which might be less developed or stable in juvenile mice.

The results of the tube dominance test in Study 1 revealed greater assertiveness of male HOM mice than male WT mice but no significant effect in females. In Study 2, male and female HOM mice were both more assertive than WT mice. Greater assertiveness of HOM mice on the tube dominance test is unexpected given the reported association of SAPAP3 deletion with lower sociability,^32^ although it should be noted that in Study 1 of the current project, genotype-associated impairment of social interaction was not observed in SAPAP3-KO mice. A relationship of winning on the tube dominance test to activity on the OFT and anxiety-like behavior on the EPM is suggested by the results of Study 2 but not those of Study 1, so that this relationship remains an open question.

Buried food seeking is a frequently used test in olfaction research in mice.^33,34^ The Buried Oreo test used in our studies combines the predilection of mice to unearth buried food substances with the preference of mice for sweet taste. This preference is a basis for the sucrose preference test that is widely used as a measure of anhedonia in mice.^23^ In both studies reported here, HOM juvenile SAPAP3-KO mice of both sexes unearthed significantly fewer buried Oreo cookies than WT mice. There was no difference between genotypes in either study in the number of unearthed Oreos that were eaten although there was a trend in this direction in Study 2. Thus, it is not entirely clear whether the lower tendency to unearth buried Oreos in the HOM mice is a manifestation of anhedonia or lack of motivation. Psilocybin treatment in Study 2 did not alter the number of Oreos unearthed or eaten.

Contrary to these positive findings, there was no effect of SAPAP3-KO genotype on the FST, reflecting the absence of depressive-like features (Study 1), on social exploration (Study 1) or on the Novel Object Recognition test (Study 1). The lack of difference on the FST, which is sensitive to activity, supports the finding that in other tests (such as EPM and MBT) the significant effect of genotype was not a consequence of reduced activity in HOM SAPAP3-KO mice. In considering these negative results, it should be borne in mind that although the order of behavioral testing was the same in Studies 1 and 2, the timing of the tests in relation to each other was not identical. It cannot be excluded that proximity in time of certain tests may influence test outcome. The timing of the tests could also have influenced the response to PSIL in Study 2.

Our findings reveal significant effects of genotype on synaptic plasticity markers in adult SAPAP3-KO mice in the frontal cortex, amygdala, hippocampus, and striatum. In post hoc analyses by sex, the synaptic proteins showing significantly higher levels in male HOM mice were GAP43 (in frontal cortex and amygdala), synaptophysin (in frontal cortex), and SV2A (in hippocampus, amygdala, and striatum). In female HOM mice, SV2A was significantly elevated in the frontal cortex compared to female WT mice. Although there was a significant effect of genotype on PSD95 in the frontal cortex, levels of this synaptic protein were not significantly different by post hoc testing in either sex separately in any of the 4 brain areas. No significant effects of genotype or sex on synaptic protein levels were observed in juvenile SAPAP3-KO mice aged 12 weeks. Our findings regarding genotype are partially in accordance with those of Glorie et al.,^35^ who used positron emission tomography to examine SV2A levels in the cortex, hippocampus, thalamus, and striatum of female SAPAP3-KO and WT mice. Glorie et al.^35^ found that at the age of 3 months, the female SAPAP3-KO mice they studied manifested significantly lower SV2A binding compared to WT littermates in the cortex and hippocampus, thalamus, and striatum. Aging in WT mice was associated with a significant (*P* < .001) decline in SV2A binding throughout the brain, whereas in SAPAP3-KO mice, this decline was confined to the cortico-striatal level.^35^

The increase in synaptic proteins in SAPAP3-KO mice points to enhanced synaptic growth and vesicle-associated plasticity in the affected regions. GAP43 is a marker of axonal growth and synaptic remodeling, while synaptophysin and SV2A are essential components of synaptic vesicle regulation and neurotransmitter release.^36-38^ SAPAP3 is a scaffold protein that interacts directly with PSD95, as its name—SAP90/PSD95-associated protein 3—suggests. Importantly, SAPAP3 is a postsynaptic protein located in the postsynaptic density and is highly expressed in the striatum. Despite this, our findings showed less striking changes in PSD95 levels in male or female adult KO mice. This suggests that the alterations we observed may be secondary to disrupted signaling pathways involving the cortico-striatal-thalamic-cortical (CSTC). Specifically, the absence of SAPAP3 may disrupt its interaction with PSD95, a key protein in the postsynaptic density, impairing **α-amino-3-hydroxy-5-methyl-4-isoxazolepropionic acid** (AMPA)receptor trafficking.^39^ This postsynaptic disruption would lead to altered excitatory synaptic transmission in the cortico-striatal circuit, a brain region critical for behavior regulation and implicated in OCD.^1,40^ Dysregulated postsynaptic function in adult SAPAP3-KO mice may trigger compensatory presynaptic changes in synaptic organization through specific increases in proteins such as GAP43, synaptophysin, and SV2A, as was observed. These changes appear age-dependent, as synaptic alterations may differ in juvenile mice, potentially due to ongoing brain maturation and synaptic pruning.^41^ Thus, the altered plasticity markers we observed are likely secondary to disrupted signaling pathways in the CSTC circuit, contributing to the behavioral phenotype in adult SAPAP3-KO mice. The increased synaptic proteins in the cortex, hippocampus, and amygdala of male SAPAP3-KO mice could reflect compensatory changes in response to dysregulated inputs from the striatum.^42^ Thus, plasticity alterations in SAPAP3-KO mice may be presynaptic, affecting synaptic transmission and connectivity rather than postsynaptic structures. This is intriguing given that synaptic pruning, a normal developmental process that refines neural circuits during adolescence involves the selective removal of unnecessary synapses, typically reducing plasticity in adult brain regions.^43^ In this context, it is of interest that Glorie et al.^35^ found a decrease in SV2A binding in adults compared to juvenile SAPAP3-WT mice. This decrease was less consistent in SAPAP3-KO mice.

As noted, the observed increases in synaptic proteins occurred only in adult SAPAP3-KO mice. The fact that these changes were absent in younger mice supports the idea of an age-dependent progression of pathology, potentially related to abnormal synaptic pruning. In healthy humans, synaptic pruning during adolescence serves to eliminate excess synapses, optimizing neural networks for efficient processing.^43^ Disruptions in this process have been implicated in various neuropsychiatric disorders.^44,45^ Specifically, delayed or abnormal pruning has been associated with atypical neural development, as seen in humans with higher neuropsychopathological factor scores, where a reduction in gray matter volume is inhibited during adolescence.^46^ These findings suggest that similar processes may occur in male SAPAP3-KO mice, with altered synaptic pruning contributing to the pathological increase in synaptic proteins in adulthood. The increase in plasticity markers in older SAPAP3-KO mice might reflect a compensatory or maladaptive response to impaired pruning, particularly in regions involved in emotional and cognitive regulation. This may explain why the obsessive grooming behaviors, a hallmark of this model, emerge in adulthood rather than earlier in life.

Despite molecular differences, male and female SAPAP3 KO mice showed similar OCD-like behaviors,^3^ indicating that behavioral phenotypes were not strongly influenced by sex. The less striking synaptic protein increases in females, despite comparable behaviors, may be due to sex-specific neurodevelopmental processes. Studies show distinct patterns of synaptic pruning and plasticity between sexes, influenced by hormones like testosterone and estrogen, which shape brain plasticity differently.^47^ Males undergo more robust synaptic pruning in regions like the nucleus accumbens during adolescence,^43^ possibly explaining the observed increase in plasticity markers in older male mice.

## CONCLUSIONS

Our findings demonstrate, in 2 separate mouse cohorts, high levels of anxiety-like behavior in juvenile SAPAP3-KO mice that are HOM for deletion of the gene, as well as other behavioral differences. The high levels of anxiety-like behavior that we observed were not responsive to treatment with PSIL. The excessive self-grooming observed in adult SAPAP3-KO mice is regarded as possibly modeling OCD; our current findings are analogous to the prodromal anxiety observed in patients with OCD and can provide an important basis for further studies. Our intriguing findings regarding increased neuroplasticity in adult, but not juvenile, male SAPAP3-mice are a basis for further research to elucidate the mechanisms underlying these changes and their implications for OCD.

## Supplementary Material

pyaf022_suppl_Supplementary_Materials

## Data Availability

Data are available to qualified investigators on request from the corresponding authors.
